# QTL mapping for quality traits using a high-density genetic map of wheat

**DOI:** 10.1371/journal.pone.0230601

**Published:** 2020-03-24

**Authors:** Ying Guo, Guizhi Zhang, Baojin Guo, Chunyan Qu, Mingxia Zhang, Fanmei Kong, Yan Zhao, Sishen Li

**Affiliations:** 1 State Key Laboratory of Crop Biology / Shandong Key Laboratory of Crop Biology, Shandong Agricultural University, Tai’an, Shandong, China; 2 Cotton research center, Shandong Academy of Agricultural Sciences, Jinan, Shandong, China; 3 Zaozhuang University, Zaozhuang, Shandong, China; North Dakota State University, UNITED STATES

## Abstract

Protein- and starch-related quality traits, which are quantitatively inherited and significantly influenced by the environment, are critical determinants of the end-use quality of wheat. We constructed a high-density genetic map containing 10,739 loci (5,399 unique loci) using a set of 184 recombinant inbred lines (RILs) derived from a cross of ‘Tainong 18 × Linmai 6’ (TL-RILs). In this study, a quantitative trait loci (QTLs) analysis was used to examine the genetic control of grain protein content, sedimentation value, farinograph parameters, falling number and the performance of the starch pasting properties using TL-RILs grown in a field for three years. A total of 106 QTLs for 13 quality traits were detected, distributed on the 21 chromosomes. Of these, 38 and 68 QTLs for protein- and starch-related traits, respectively, were detected in three environments and their average values (AV). Twenty-six relatively high-frequency QTLs (RHF-QTLs) that were detected in more than two environments. Twelve stable QTL clusters containing at least one RHF-QTL were detected and classified into three types: detected only for protein-related traits (type I), detected only for starch-related traits (type II), and detected for both protein- and starch-related traits (type III). A total of 339 markers flanked with 11 QTL clusters (all except C6), were found to be highly homologous with 282 high confidence (HC) and 57 low confidence (LC) candidate genes based on IWGSC RefSeq v 1.0. These stable QTLs and RHF-QTLs, especially those grouped into clusters, are credible and should be given priority for QTL fine-mapping and identification of candidate genes with which to explain the molecular mechanisms of quality development and inform marker-assisted breeding in the future.

## Introduction

Common wheat (*Triticum aestivum* L., 2n = 6x = 42, AABBDD) is one of the most widely grown crops worldwide, with an annual output of more than 620 million tonnes. As a staple food crop for 30% of the human population, approximately 20% of the protein and calories consumed by humans come from wheat (FAO, http://www.fao.org/faostat/). Food products, such as bread, cake, noodles and steamed bread, made from processed wheat grain are consumed daily across the globe. Improving processing and end use quality is an important objective in wheat breeding programs [1, 2].

In wheat, protein and starch functionality are the critical determinants of end-use quality. Protein- and starch-related quality traits include grain protein content, dough rheological properties, sedimentation value, falling number (FN), and starch pasting characteristics. Grain protein content can affect the nutritional value and baking properties of wheat, which also influence the dough rheological properties and are critical marketing characteristics [3, 4]. Rheological properties govern the performance of wheat flour dough during mechanical treatment [5, 6]. Farinograph parameters, including water absorption (WA), development time (DT), and stability time (ST), are used to assess these rheological properties, providing insight into baking performance [7]. The sedimentation value is measured to evaluate the gluten strength, which is correlated with dough rheological properties [8]. The falling number is an important quality trait that determines the effect of ɑ-amylase on wheat grain starch [9–11]. Starch pasting properties are highly related to the quality of steamed bread, noodles, and other products [12].

Quality traits are quantitatively inherited and significantly influenced by the environment [12]. Quantitative trait locus (QTL) analysis based on high-density genetic maps provides an effective approach to dissect complicated traits into component loci and study the relative effects of the loci on a specific trait [13]. In recent decades, multiple QTL mapping studies have been performed to understand the genetic architecture underlying end-use quality, including grain protein content [2; 8; 12; 14–20], dough rheological properties [2; 12; 19; 21; 22], sedimentation value [4; 23–26], falling number [11; 27; 28] and starch pasting properties [24; 29–32]. Some stable or relatively high frequency QTLs (RHF-QTL) and important QTL clusters in the same genomic regions have been detected. For example, McCartney et al. [23] identified 99 QTLs over 18 chromosomes for 41 quality traits. One stable locus, which included twenty of those QTLs, was mapped on chromosome 4D. Another 10 QTLs, which were mainly associated with traits for mixograph and farinograph performance, baking performance, and starch functionality, were mapped near a QTL for time to maturity on chromosome 7D. Mann et al. [2] detected two relatively high frequency QTLs for grain protein content on chromosomes 3A and 7A at three of five sites, and two dough rheology QTLs were highly consistent across the 5 sites, with major effects associated with the *Glu-B1* and *Glu-D1* loci identified. Carter et al. [8] identified two QTL clusters located on chromosomes 3B and 4D and associated with traits for milling quality and starch functionality. Boehm Jr. et al. [18] identified four stable genomic regions on chromosomes 1BL, 2DS, 7AS and 7BS, with numerous colocalizing QTLs influencing multiple end-use quality traits, such as grain protein content, water absorption, and flour yield.

Single nucleotide polymorphisms (SNPs), the discovery of which was largely based on sequence information, are abundant, codominant and uniform throughout the genome [33]. Recently, SNP genotyping arrays, such as the 90K iSelect and 660K SNP array, have been used to construct high-density maps and detect QTLs in wheat [31; 34–39]. High-density genetic maps are useful in detecting QTLs related to important agronomical traits, identifying and localizing candidate genes, and map-based cloning of genes of interest and in marker-assisted selection (MAS) and predictive molecular breeding [33; 40; 41].

In this study, we performed QTL analysis of 13 quality traits measured with the seeds sample harvested from the three growing season and their mean values using a high-density genetic map containing 10,739 loci (5,399 unique loci) covering all 21 chromosomes [42]. The aims of the present study were to identify QTLs for quality traits using the RIL population and find relatively stable QTL and QTL clusters, which may be used in QTL cloning and wheat breeding programs.

## Materials and methods

### Plant materials and field trials

The RIL population used in the study was derived by single-seed descent (SSD) methods from a cross of “Tainong18 × Linmai6” (TL-RIL, F_8_ in 2013). Tainong18 (TN18) is a cultivated variety developed by our research group (Approved Number: Lunongshen 2008056), which was released in 2008 and is planted on approximately 30 thousand hectares per year in the Huang-huai winter wheat region, China. TN18 possesses several salient features, such as dwarfness and excellent lodging resistance, high grain yield and fine quality. TN18 became a core parent with the development of the cultivars Shannong 29 (Approved Number: Lunongshen 2016002, Guoshenmai 2016024) and Shannong 30 (Approved Number: Guoshenmai 20170019) and over 20 strains under regional trials. The male parent Linmai6 (LM6) is an elite wheat line developed by the Linyi Academy of Agricultural Science, the mother of which is a sister line of the famous cultivar “Jimai 22” (Approved Number: Lunongshen 2006050, Guoshenmai 2006018). A total of 184 lines of the RILs, which were randomly selected from the original 305 lines, were used to construct the genetic map and investigate the phenotypes.

The field trials were conducted at the experimental farm of the Shandong Agricultural University in Tai’an for three growing seasons with two replications: 2011–2012 (E1), 2012–2013 (E2), and 2013–2014 (E3). The seeds of the TL-RILs and their parents were sown in early October every year and harvested in early June the next year. Each plot consisted of 3 rows that were 1.2 m long and spaced 25 cm apart. Fifty seeds were planted in each row. The experimental fields had loamy soil, and the grain yield was approximately 9 000 kg·ha^−1^. During the growing season, cultural management were carried out according to the local cultivation practices and the plants were not affected by insect pests, disease, or lodging, and the rainfalls in Tai’an was 190–220 mm.

### Quality trait measurements

The seed samples obtained from the harvested populations were stored at room temperature for approximately three months and then milled using a Bühler experimental mill (Bühler mill, Bühler-Miag Company, Braunschweig, Germany). The whole flour extracted from the wheat grain with natural drying, was obtained with a Perten 3100 experimental mill (Perten Instruments AB, Huddinge, Sweden). Meanwhile the moisture content of whole flour was measured by direct drying method according to GB/T 5009.3–2016, which was used to adjust the GPC values based on dry matter. The flour extraction rate was approximately 70%, and the flours were stored at 4°C until use. The whole flour was used to test the grain protein content (GPC), and the flour was used to test the sedimentation value (SV), farinograph parameters, falling number (FN) and starch pasting properties. The GPC was measured by the Kjeldahl method using an NC analyzer (KDY-9820, Tongrun Ltd., China). The SV was determined with a sedimentation volume instrument (BAU-A type) [30]. The farinograph parameters, including development time (DT), stability time (ST), and water absorption (WA), were determined by a farinograph (Brabender GmbH and Co KG). The FN was determined using a falling number instrument (FN1500 type, Perton Co., Sweden). The starch pasting properties, including peak viscosity (PV), trough viscosity (TV), final viscosity (FV), breakdown (BD), setback (SB), peak time (PTi) and pasting temperature (PTe), were measured with a Rapid ViscoAnalyser (RVA-Super 3, Newport Scientific, Australia), following the manufacturer’s instructions.

### Data analysis

The analyses of variance (ANOVA) were calculated using SPSS 17.0 software (SPSS Inc., Chicago, IL, USA) and Excel 2010. Pearson correlations coefficients (*r*) between different traits under each environment were calculated and plotted in R version 3.2.2 (https://www.r-project.org/). The frequency distribution of different traits was also plotted using R software. Heritability was calculated using a model in which the three environments were regarded as three replications, and the genotype × treatment interaction was used as the error term. The broad-sense heritability (*h*_*B*_^2^) was estimated according to the following formula: *h*_*B*_^*2*^ *= σ*_*g*_^*2*^*/ (σ*_*g*_^*2*^*+σ*_*e*_^*2*^*)*, where *σ*_*g*_^*2*^ is the genotypic variance and *σ*_*e*_^*2*^ is the total error variance.

### The high-density genetic map

The TL-RILs and their parents were scanned with a total of 156 thousand markers, including the wheat *Pst*I (*Taq*I) V3 and wheat GBS1.0 DArT arrays (Diversity Arrays Technology, https://www.diversityarrays.com/), the iSelect 90K SNP array [34], and SSR (http://wheat.pw.usda.gov) and EST-SSR markers [43–45]. The anchor information for the SSR markers was obtained from the GrainGene website (http://wheat.pw.usda.gov), and the anchor information for the DArT was provided by DArT arrays canning results (https://www.diversityarrays.com/) and SNP arrays was provided by Wang et al [34]. A linkage analysis was performed in two steps using JoinMap^®^ 4.0 [46]. The map construction was performed using the Kosambi mapping function with the following JoinMap parameter settings: Rec = 0.35, LOD = 4.0 and Jump = 5. A regression mapping algorithm was used to order the markers in the maps and to calculate the genetic distances between the markers. The linkage groups were named according to the wheat chromosome nomenclature, followed by a number. Among the 184 RILs, the markers with identical genotypes were defined as cosegregated and considered unique loci, which were named using randomly selected marker names. Finally, 10 739 markers were mapped to the genetic map, including 5548 DArTs, 5085 SNPs, and 106 SSR and EST-SSRs (S1 Table). Eliminating the colocated markers, the whole map consisted of 5399 unique loci, including 3788 DArTs, 1506 SNPs, and 105 SSRs and EST-SSRs. The genetic map spanned a total map length of 3394.47 cM and included 43 linkage groups covering all 21 chromosomes, with an average chromosome length of 161.64 cM and a marker density of 0.63 cM/marker (S1 Table).

### QTL mapping

Both the phenotypic values obtained from the three environments (E1, E2, and E3) and their average values (AV) were used for QTL mapping analyses. Windows QTL Cartographer 2.5 software [47] was used to perform the QTL mapping. Composite-interval mapping (CIM) was selected to search for QTL of each trait separately. The parameter setup ‘‘model 6 standard analysis” was used with a walk speed of 0.5 cM, ‘‘forward and backward” regression for the selection of the markers to control for the genetic background, up to five control markers, and a blocked window size of 10 cM to exclude closely linked control markers at the testing site. The threshold for declaring the presence of a significant QTL was defined by 1,000 permutations at *p*≤0.05 [48] and the minimum LOD score was chosen. We defined the relatively high frequency QTL (RHF-QTL), which was detected in more than two environments. A QTL cluster was defined as two or more traits with significant QTLs (at least one RHF-QTL contained) having overlapping confidence intervals.

### Identification of candidate genes related to QTLs

To determine whether these flanking chromosomal region markers are associated with candidate genes for quality traits, all the marker sequences from the QTL cluster confidence interval were aligned with the wheat reference genome (IWGSC RefSeq v1.0). In addition, according to the results of the alignment of the genetic linkages and physical maps based on IWGSC RefSeq v1.0, we predicted all the candidate genes within the confidence intervals associated with the 12 QTL clusters for quality traits. BLAST analyses were carried out on Chinese Spring IWGSC RefSeq v1.0 using tools available at the URGI (https://wheat-urgi.versailles.inra.fr/Tools/BLAST-Public). Gene annotations were obtained from https://wheat-urgi.versailles.inra.fr/Seq-Repository/Annotations.

## Results

### Phenotypic variation and correlation analysis

The parents of the RIL population, TN18 and LM6, showed obvious differences for most of the investigated traits (Table 1). TN18 had higher SV, DT, ST than LM6, but lower GPC, WA, and starch pasting properties except SB than LM6. For the RIL population, there was a wide range of variation with from 1.35% to 47.48% (Table 1). Transgressive segregation was observed for almost all of the quality traits under E1, E2, E3 and AV. All 13 investigated traits in each environment (including AV) exhibited a continuous distribution (S1 Fig). The *h*_*B*_^*2*^ for the investigated traits ranged from 29.23 (Pte) to 70.05% (SV) (Table 1). The results of the ANOVA showed that the variance of the genotype and the environmental effects of the 13 investigated traits were significant at *p* ≤ 0.001 (Table 2).

**Table 1:** Phenotypic performance of the TL-RILs and their parents under the E1, E2, E3 and AV environments.

Traits ^a^	Treatments	Parents ^b^		TL-RILs (n = 184)					*h*_*B*_^*2*^ (%) ^e^
		TN18	LM6	AV	SD ^c^	CV (%) ^d^	Max	Min	
GPC	E1	14.35	15.49	13.93	0.75	5.41	16.07	12.14	43.62
	E2	14.05	15.81	14.15	0.92	6.49	16.69	11.21	
	E3	12.72	15.78	13.76	1.30	9.42	19.40	10.70	
	AV	13.71	15.69	13.94	0.72	5.20	16.00	11.97	
SV	E1	26.60	25.40	27.05	2.55	9.42	33.80	20.10	70.05
	E2	25.60	24.80	28.11	2.84	10.09	36.80	23.50	
	E3	29.60	26.80	29.59	3.74	12.63	41.70	21.10	
	AV	27.27	25.67	28.30	2.64	9.34	36.95	22.17	
DT	E1	5.70	3.20	4.30	1.23	28.72	10.30	2.20	48.14
	E2	3.05	3.35	3.24	1.07	33.00	8.80	1.50	
	E3	5.20	2.67	3.74	1.14	30.48	6.90	1.90	
	AV	4.65	3.07	3.74	0.85	22.64	6.91	1.95	
ST	E1	8.00	2.30	6.68	2.93	43.90	19.00	2.80	69.02
	E2	8.40	2.50	8.20	3.84	46.83	17.90	2.60	
	E3	6.20	2.55	5.82	2.76	47.48	14.81	1.50	
	AV	7.53	2.45	6.93	2.71	39.10	14.70	2.67	
WA	E1	57.60	61.70	58.90	1.39	2.36	62.60	55.80	67.28
	E2	57.25	60.60	58.53	1.50	2.57	61.70	55.50	
	E3	60.90	63.20	61.76	2.26	3.66	66.80	47.30	
	AV	58.58	61.83	59.76	1.51	2.53	63.40	53.37	
FN	E1	618.00	625.00	638.69	39.92	6.25	759.00	524.00	40.08
	E2	487.50	451.50	523.41	46.31	8.85	665.00	396.00	
	E3	504.00	434.00	495.73	33.92	6.84	562.00	368.00	
	AV	536.50	503.50	549.59	31.42	5.72	643.33	442.00	
PV	E1	174.33	193.92	176.50	14.27	8.09	258.71	147.50	52.03
	E2	155.98	184.06	176.85	19.03	10.76	224.58	119.54	
	E3	137.13	166.63	147.19	15.99	10.86	209.54	88.42	
	AV	155.81	181.53	166.26	12.65	7.61	211.26	133.96	
TV	E1	137.42	143.33	139.58	11.72	8.39	197.96	113.96	49.07
	E2	91.54	109.44	114.71	17.72	15.45	154.25	64.38	
	E3	111.71	115.75	116.51	15.03	12.90	155.54	44.42	
	AV	113.56	122.84	123.00	11.52	9.36	154.06	91.19	
FV	E1	227.88	228.83	233.12	16.56	7.11	314.92	175.96	49.19
	E2	187.75	203.94	217.23	24.69	11.37	266.63	139.21	
	E3	197.79	206.46	209.84	21.23	10.12	259.58	118.50	
	AV	204.47	213.08	219.54	15.67	7.14	254.94	177.25	
BD	E1	36.92	50.58	37.30	6.00	16.09	62.88	24.96	51.89
	E2	64.44	74.63	62.14	5.24	8.44	73.83	44.88	
	E3	25.42	50.88	30.68	6.59	21.48	54.00	17.25	
	AV	42.26	58.69	43.27	5.32	12.30	58.61	25.15	
SB	E1	90.46	85.50	94.21	5.68	6.03	117.08	79.38	44.08
	E2	96.21	94.50	102.51	8.34	8.13	118.83	74.63	
	E3	86.08	90.71	93.33	7.75	8.30	113.54	72.88	
	AV	90.92	90.24	96.65	5.25	5.44	107.85	83.36	
Pti	E1	6.50	6.60	6.51	0.09	1.35	6.73	6.27	48.18
	E2	6.10	6.27	6.23	0.15	2.39	6.57	5.73	
	E3	6.43	6.43	6.41	0.14	2.13	6.67	5.57	
	AV	6.34	6.43	6.38	0.10	1.55	6.61	6.07	
Pte	E1	64.90	64.03	63.87	1.16	1.82	70.15	59.80	29.23
	E2	63.03	63.98	61.67	1.33	2.16	64.48	58.08	
	E3	65.70	65.75	65.04	2.12	3.26	73.35	58.08	
	AV	64.54	64.58	63.54	1.04	1.63	66.98	60.74	

^a^ GPC, grain protein content; SV, sedimentation volume; FN, falling number; WA, water absorption; DT, development time; ST, stability time; PV, peak viscosity; TV, trough viscosity; BD, breakdown; FV, final viscosity; SB, setback; PTi, peak time; PTe, pasting temperature.

^b^ TN18, Tainong 18; LM6, Linmai 6.

^c^ SD, Standard deviation.

^d^ CV, (Coefficient of variation, %) = SD/average * 100.

^e^ *h*_*B*_^*2*^(%), broad-sense heritability.

**Table 2:** Analysis of variance (ANOVA) for the investigated quality traits.

Traits	Source of variation	
	Genotypes	Environments
GPC	4.09***	8.00***
SV	10.36***	61.89***
DT	4.71***	42.39***
ST	9.91***	58.13***
WA	9.23***	350.80***
FN	3.68***	546.21***
PV	5.34***	270.43***
TV	4.85***	168.91***
FV	4.87***	70.93***
BD	5.31***	1416.67***
SB	4.15***	104.31***
Pti	4.72***	255.12***
Pte	2.65***	199.62***

*** indicates significance at *p*≤ 0.001

**Fig 1:**
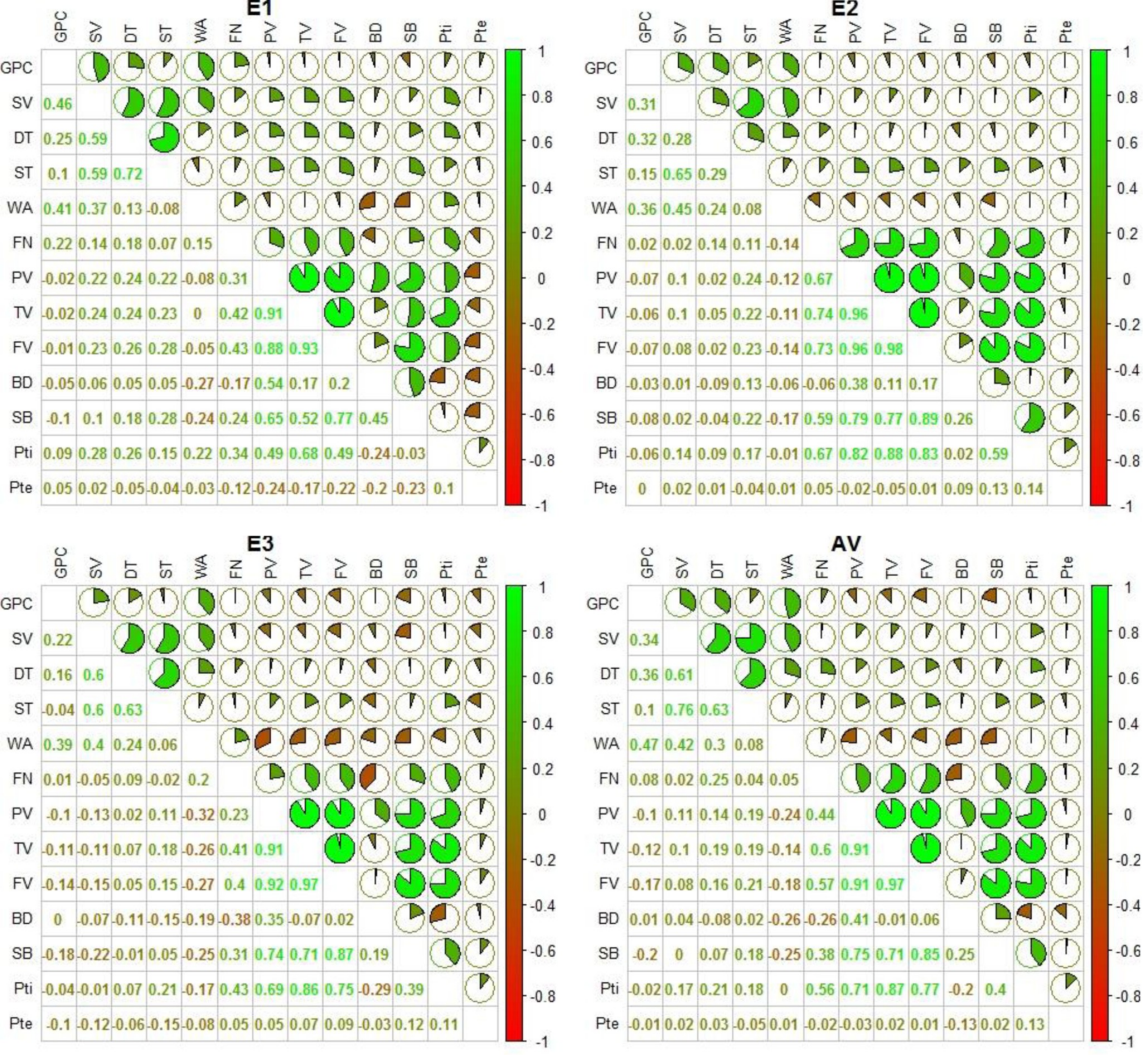
The pearson correlation coefficients (*r*) between investigated quality traits under E1, E2, E3 and AV environments.

Most pearson correlation coefficients (*r*) among protein-related traits (GPC, SV, WA, DT and ST) were significant, except for the *r* between ST and GPC, between WA and ST (Fig 1). Likewise, most pearson correlation coefficients (*r*) among the starch-related traits (FN, PV, TV, FV, BD, SB, PTi and PTe) were significant, except for the *r* between BD and TV, between Pte and FN/PV/TV/FV/BD/SB/PTi (Fig 1). In addition, the *r* values between the protein- and starch-related traits were relatively less significant; for example, the GPC was significantly negatively correlated with the FV and SB traits, the SV was significantly correlated with the PTi traits, the WA was significantly negatively correlated with the PV, BD, FV and SB traits, the DT was significantly correlated with the FN, TV, FV and PTi traits, and the ST was significantly correlated with the TV, FV, SB and PTi traits (Fig 1).

### QTL analysis

**Fig 2:**
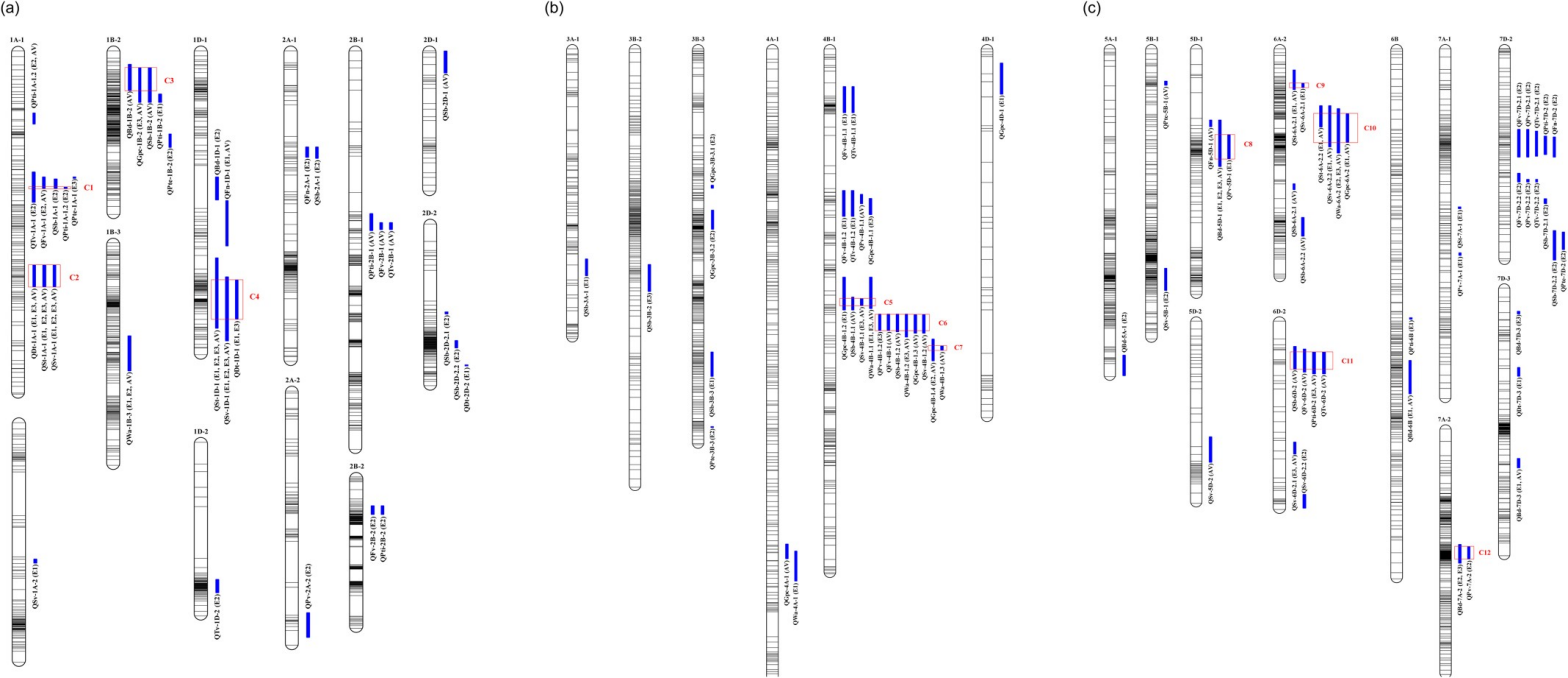
Locations of QTLs for 13 quality traits based on RILs derived from TN18 × LM6. QTL intervals were LOD ≥ 3.0 with LOD peak values more than threshold values, which were determined by 1000 permutation test (*p*≤0.05). The QTLs with *blue* on the figure refer to the QTLs for quality traits.

According to the results of 1,000 permutations at *p*≤0.05, the minimum LOD score for each trait-environment is listed in S2 Table. A total of 106 QTLs for 13 quality traits were detected, distributed on the 21 chromosomes (S3 Table; Fig 2). The QTLs explained 5.32–35.09% of the phenotypic variation. The highest LOD value for a single QTL was 30.2.

#### QTL for the protein-related traits

For the GPC, 10 QTLs were detected on chromosomes 1B, 3B, 4A, 4B, 4D and 6A (S3 Table; Fig 2). Each individual QTL explained between 7.08% (*QGpc-3B-3*.*1* in E2) and 15.52% (*QGpc-1B-2* in E3) of the phenotypic variation. The highest LOD value for a single QTL was 7.6, which was observed at *QGpc-1B-2*. Among the QTLs, five showed positive additive effects, with TN18 increasing the effects of the QTLs, while five had negative additive effects, with LM6 increasing the effects of the QTLs. Eleven QTLs for the SV were detected on chromosomes 1A, 1D, 4B, 5B, 5D, 6A and 6D (S3 Table; Fig 2). Each individual QTL explained between 5.32% (*QSv-4B-1*.*2* in AV) and 18.73% (*QSv-6D-2*.*2* in E2) of the phenotypic variation. The highest LOD value for a single QTL was 13.6, which was observed at *QSv-6D-2*.*2*. Among the QTLs for the SV, four showed positive additive effects, with TN18 increasing the effects of the QTLs, while seven had negative additive effects, with LM6 increasing the effects of the QTLs. For the three farinograph parameters (DT, ST and WA), five, five and seven QTLs were detected, respectively (S3 Table; Fig 2). Each individual QTL explained between 5.65% (*QSt-7A-1* in E1) and 35.09% (*QSt-1D-1* in AV) of the phenotypic variation. The highest LOD value for a single QTL was 30.2, which was observed at *QSt-1D-1*. Among the QTLs for the farinograph parameters, nine showed positive additive effects, with TN18 increasing the effects of the QTLs, while eight had negative additive effects, with LM6 increasing the effects.

#### QTL for the starch-related traits

For the FN, four QTLs were detected on chromosomes 1D, 2A, 5D and 7D (S3 Table; Fig 2). Each individual QTL explained between 8.43% (*QFn-2A-1* in E2) and 10.71% (*QFn-7D-2* in E2) of the phenotypic variation. The highest LOD value for a single QTL was 5.9, which was observed at *QFn-7D-2*. Among the QTLs for the FN, all four showed negative additive effects, with LM6 increasing the effects of the QTLs. For the seven starch pasting properties (BD, FV, Pte, Pti, PV, SB, TV), eight, nine, six, nine, eight, sixteen, and eight QTLs were detected, respectively (S3 Table; Fig 2). Each individual QTL explained between 6.30% (*QPti-6D-2* in AV) and 17.90% (*QPti-2B-1* in AV) of the phenotypic variation. The highest LOD value for a single QTL was 10.6, which was observed at *QPti-2B-1*. Among the QTLs for the starch pasting properties, twenty-five showed positive additive effects, with TN18 increasing the effects of the QTLs, while thirty-nine had negative additive effects, with LM6 increasing the effects of the QTLs.

#### RHF-QTLs and QTL clusters

For the protein-related traits, a total of 18 RHF-QTLs were detected: *QGpc-1B-2*, *QGpc-4B-1*.*4*, *QGpc-6A-2*, *QSv-1A-1*, *QSv-1D-1*, *QSv-4B-1*.*1*, *QSv-6A-2*.*2*, *QSv-6D-2*.*1*, *QDt-1A-1*, *QDt-1D-1*, *QSt-1A-1*, *QSt-1D-1*, *QSt-6A-2*.*1*, *QSt-6A-2*.*2*, *QWa-1B-3*, *QWa-4B-1*.*1*, *QWa-4B-1*.*2*, *and QWa-6A-2* (Table 3, Fig 2). Among these RHF-QTLs, 11 showed positive additive effects, with TN18 increasing the effects of the QTLs, while seven had negative additive effects, with LM6 increasing the effects of the QTLs. For the starch-related traits, eight RHF-QTLs were detected: *QFn-1D-1*, *QBd-5D-1*, *QBd-6B*, *QBd-7A-2*, *QBd-7D-3*, *QFv-1A-1*, *QPti-1A-1*.*2*, *and QPti-6D-2* (Table 3, Fig 2). Among these RHF-QTLs, three showed positive additive effects, with TN18 increasing the effects of the QTLs, while five had negative additive effects, with LM6 increasing the effects of the QTLs.

**Table 3:** Summary of the relatively high frequency QTLs (RHF-QTLs) detected in more than two environments.

Traits	QTL	Environments	Marker interval ^a^	LOD			Additive effect ^b^			*R*^*2*^ (%)		
				MAX	MIN	AV	MAX	MIN	AV	MAX	MIN	AV
GPC	*QGpc-1B-2*	E3, AV	*D-1190331~S-3222160*	7.6	5.5	6.5	0.5269	0.2310	0.3789	15.52	9.47	12.49
	*QGpc-4B-1*.*4*	E2, AV	*D-1380792~D-1094306*	7.3	5.3	6.3	0.2932	0.2756	0.2844	14.09	9.82	11.95
	*QGpc-6A-2*	E1, AV	*D-1112857~S-2362461*	6.2	5.7	6.0	-0.2532	-0.3037	-0.2785	10.66	9.63	10.15
SV	*QSv-1A-1*	E1, E2, E3, AV	*S-1089822~S-3953635*	10.3	3.9	8.3	1.0588	0.9003	0.9818	16.31	5.71	11.63
	*QSv-1D-1*	E1, E2, E3, AV	*D-1234123~S-1225816*	10.4	3.9	7.1	1.5855	0.6057	0.9612	17.16	5.45	10.12
	*QSv-4B-1*.*1*	E3, AV	*S-1040960~D-1083795*	5.2	4.6	4.9	0.9884	0.6630	0.8257	6.76	6.15	6.46
	*QSv-6A-2*.*2*	E1, AV	*D-1112857~D-1092061*	10.7	4.6	7.6	-0.6668	-1.0493	-0.8580	16.75	5.40	11.08
	*QSv-6D-2*.*1*	E3, AV	*S-1023247~D-4329585*	5.4	4.4	4.9	-0.8762	-0.9894	-0.9328	9.71	6.65	8.18
DT	*QDt-1A-1*	E1, E3, AV	*S-1000608~Kukri_c37726_285*	8.5	5.9	7.1	0.4685	0.2948	0.3795	14.01	10.62	12.11
	*QDt-1D-1*	E1, E3	*D-1170288~D-2256216*	7.8	5.1	6.4	0.4493	0.3692	0.4092	12.53	9.46	10.99
ST	*QSt-1A-1*	E1, E2, E3, AV	*D-1207393~S-1000608*	19.0	5.0	10.5	1.5287	0.7335	1.0728	18.84	6.78	12.19
	*QSt-1D-1*	E1, E2, E3, AV	*D-1122483~D-2256216*	30.2	16.5	20.9	2.0563	1.5335	1.6938	35.09	24.85	28.96
	*QSt-6A-2*.*1*	E1, AV	*wPt-731556~D-3952327*	10.4	4.6	7.5	-0.8538	-0.9568	-0.9053	9.39	9.21	9.30
	*QSt-6A-2*.*2*	E1, AV	*BS00066274_51~D-1219492*	7.1	4.9	6.0	-0.7325	-0.7395	-0.7360	7.23	6.27	6.75
WA	*QWa-1B-3*	E1, E2, AV	*D-1134712~S-1027932*	12.9	6.2	9.2	-0.4630	-0.7119	-0.5729	20.37	7.87	14.19
	*QWa-4B-1*.*1*	E1, E3, AV	*D-3022151~S-1040960*	15.7	10.2	12.4	1.0124	0.7305	0.8547	28.76	19.51	25.10
	*QWa-4B-1*.*2*	E3, AV	*D-1138250~D-3943712*	10.4	5.2	7.8	0.8123	0.7523	0.7823	18.13	9.21	13.67
	*QWa-6A-2*	E2, E3, AV	*D-1026376~Excalibur_rep_c69981_75*	6.7	5.4	5.8	-0.4153	-0.6631	-0.5234	10.34	7.47	8.70
FN	*QFn-1D-1*	E1, AV	*cfd19~D-1073588*	5.3	4.5	4.9	-10.1489	-12.6569	-11.4029	9.73	9.55	9.64
FV	*QFv-1A-1*	E2, AV	*D-3385037~S-3030753*	6.9	3.8	5.3	8.5222	4.0388	6.2805	11.46	6.31	8.89
BD	*QBd-5D-1*	E1, E2, E3, AV	*S-1862723~D-1055236*	9.0	4.6	7.0	2.6700	1.5379	2.0613	15.96	8.20	12.19
	*QBd-6B*	E1, AV	*D-1100407~S-1054930*	6.3	5.6	5.9	-1.7621	-1.9717	-1.8669	10.64	10.57	10.60
	*QBd-7A-2*	E2, E3	*S-987549~D-1127555*	6.9	4.7	5.8	-1.5677	-4.0067	-2.7872	13.48	8.34	10.91
	*QBd-7D-3*	E1, AV	*wPt-732048~D-1072335*	6.2	5.1	5.7	-1.6852	-1.8725	-1.7788	9.67	9.33	9.50
Pti	*QPti-1A-1*.*2*	E2, AV	*D-1283455~RFL_Contig1118_65*	5.5	4.6	5.0	0.0548	0.0323	0.0435	10.59	9.84	10.21
	*QPti-6D-2*	E3, AV	*D-1129747~D-3938792*	5.4	4.1	4.8	-0.0261	-0.0471	-0.0366	11.07	6.30	8.69

^a^ Marker interval means the interval of the LOD peak value for QTLs.

^b^, Positive effect, increased effect contributed by TN18; negative effect was contributed by LM6.

Considering the QTLs detected in the AV treatments, a total of 12 QTL clusters (C1-C12) were mapped to eight chromosomes (1A, 1B, 1D, 4B, 5D, 6A, 6D, and 7A), which involved 39 out of the 106 QTLs (36.8%) for the trait-treatment combinations (Table 4, Fig 2). These clusters were related to 11 of the 13 investigated traits, with the exception of FN and PTe. Twenty-one out of the 26 RHF-QTLs (80.8%) were detected in these clusters. All these QTL clusters could be classified into three types: detected only for protein-related traits (type I, including C2, C4, C7, C9 and C10), detected only for starch-related traits (type II, including C1, C11 and C12), and detected for both protein- and starch-related traits (type III, including C3, C5, C6 and C8) (Table 4, Fig 2).

**Table 4:** QTLs detected in the same or adjacent marker regions in this paper and in previous studies.

Chromosomes	The same or adjacent markers	QTLs in this study	QTLs detected in previous studies	
			Related traits	Reference
1D	*BS00018250_51*	*QSv-1D-1 (E1*, *E2*, *E3*, *AV)*	DT	Jin et al. [31]
6A	*BS00067934_51*	*QWa-6A-2 (E2*, *E3*, *AV)*	WA	
		*QSv-6A-2*.*2 (E1*, *AV)*		
		*QDt-6A-2 (AV)*		
		*QSt-6A-2*.*2 (E1*, *AV)*		
7D	*Excalibur_c4508_1007*	*QSb-7D-2*.*1 (E2)*	BD	

## Discussion

### QTL mapping compared with that of a previous study

In the present study, a total of 107 QTLs for GPC, SV, FN, three farinograph parameters and seven starch pasting properties were detected (S3 Table). The stable loci, including 26 RHF-QTLs, were mainly located on chromosomes 1B, 4B and 6A for GPC; 1A, 1D, 4B, 6D for SV; 1A and 1D for DT; 1A, 1D and 6A for ST; 1B, 4B and 6A for WA; 1D for FN; 1A for FV; 5D, 6B, 7A and 7D for BD; and 1A and 6D for Pti. By comparing our QTL mapping results with those of previously mapped QTLs, we found that some QTLs were detected on the same or adjacent marker regions as those in previous results (Table 4). For example, the RHF-QTLs for SV were detected on chromosome 1D in all the environments tightly linked with the SNP marker *BS00018250_51*, for which there was also a detected QTL for DT reported by Jin et al. [31]. In addition, some QTLs for quality traits have been detected on the same chromosomes as those identified in previous studies, such as QTLs for GPC on 4A, 4B, 4D, and 6A [16; 30; 31], for farinograph parameters on 1B, 4A, 4B, and 6A [4; 11; 17;21; 30], for FN on 7D [11], and for starch pasting properties on 1A, 1D, 7A, and 7D [30; 31]. However, due to the very large differences in the markers used to create the genetic maps, the background of the populations and the environments of the trials, the majority of the QTLs on the same chromosome were mapped to new marker regions in the present study.

### The stable QTLs for quality traits and predication of candidate genes

In wheat, a large number of stable QTLs, especially those distributed into clusters, have been mapped in the same genomic regions [2; 8; 17; 18; 23; 31]; these regions are credible and should be the priority for fine-scale mapping and identification of candidate genes with which to elaborate on the molecular mechanisms of quality development. The QTL clusters that include stable QTLs for different traits in several environments are usually detected at the same marker interval or adjacent loci on the same chromosome, which may be due to the linkage of several genes or to the pleiotropic effects of a common gene. Therefore, these stable QTL regions have a very large significance for fine-scale gene mapping, cloning and molecular marker-assisted breeding. Moreover, with the completion of the IWGSC RefSeq v1.0 assembly, it is possible to dissect quantitative traits genetically and implement modern breeding strategies for future wheat improvement by resolving the inherent complexity of gene families related to important agronomic traits [49]. In the present study, twenty-seven RHF-QTLs were identified in more than two environments (Table 3). Twenty-one of the 26 RHF-QTLs were grouped into 12 QTL clusters (C1-C12), mapped on eight chromosomes, including 1A, 1B, 1D, 4B, 5D, 6A, 6D and 7A (Table 4, Fig 2), and classified into three types: I, II and III. By aligning with the wheat reference genome (IWGSC RefSeq v1.0), a total of 339 markers on our genetic linkage map, flanked with 11 QTL clusters (all except C6), were found to be highly homologous, with 282 high confidence (HC) and 57 low confidence (LC) candidate genes (S4 Table). In addition, according to the results of the alignment of the genetic linkages and physical maps (based on IWGSC RefSeq v1.0) (S5 Table), we predicted all the candidate genes within the confidence intervals associated with the 12 QTL clusters for quality traits (Table 5). Several important QTL clusters (e.g., C2, C3, and C4) were linked with known quality genes, and other clusters (e.g., C5, C6, and C10) could be linked with new candidate genes, which are discussed as follows.

**Table 5:** QTL clusters for more than two traits in more than two environments.

QTL cluster	Chromosome	Marker interval	Gnentic distance (cM)	No. of QTL	QTL	The physical maps based on the blast results from the IWGSC Refseq v1.0 sequence		
						Physical position (Mb)	High confidence genes	Low confidence genes
C1	1A-1	*D-3942600~D-1210851*	2.87	4	*QTv-1A-1 (E2)*	35455766~41743680	TraesCS1A01G053300~TraesCS1A01G060600	TraesCS1A01G077600LC~TraesCS1A01G084200LC
Type II					*QFv-1A-1 (E2*, *AV)*			
					*QSb-1A-1 (E2)*			
					*QPti-1A-1 (E2)*			
C2	1A-1	*BS00089894_51~wmc312*	6.07	3	*QDt-1A-1 (E1*, *E3*, *AV)*	497520605~513747786	TraesCS1A01G304400~TraesCS1A01G323400	TraesCS1A01G448200LC~TraesCS1A01G474000LC
Type I					*QSt-1A-1 (E1*, *E2*, *E3*, *AV)*			
					*QSv-1A-1 (E1*, *E2*, *E3*, *AV)*			
C3	1B-2	*D-996292~D-3024514*	6.44	3	*QBd-1B-2 (AV)*	532692753~564653616	TraesCS1B01G310100~TraesCS1B01G337300	TraesCS1B01G537600LC~TraesCS1B01G582300LC
Type III					*QGpc-1B-2 (E3*, *AV)*			
					*QSb-1B-2 (AV)*			
C4	1D-1	*swes1148~Ex_c68201_1182*	10.97	3	*QSt-1D-1 (E1*, *E2*, *E3*, *AV)*	407808667~435939122	TraesCS1D01G311700~TraesCS1D01G349800	TraesCS1D01G427300LC~TraesCS1D01G470900LC
Type I					*QSv-1D-1 (E1*, *E2*, *E3*, *AV)*			
					*QDt-1D-1 (E1*, *E3)*			
C5	4B-1	*D-3022151~D-1083795*	2.32	4	*QGpc-4B-1*.*1 (E1)*	25830709~30864754	TraesCS4B01G035300~TraesCS4B01G043100	TraesCS4B01G041000LC~TraesCS4B01G048200LC
Type III					*QWa-4B-1*.*1 (E1*, *E3*, *AV)*			
					*QSb-4B-1*.*1 (AV)*			
					*QSv-4B-1*.*1 (E3*, *AV)*			
C6	4B-1	*D-4008856~D-3943712*	4.92	6	*QPv-4B-1*.*2(E3*, *AV)*	37503144~51849569	TraesCS4B01G049300~TraesCS4B01G060000	TraesCS4B01G055300LC~TraesCS4B01G069300LC
Type III					*QFv-4B-1 (AV)*			
					*QSb-4B-1*.*2 (AV)*			
					*QWa-4B-1*.*2 (E3*, *AV)*			
					*QGpc-4B-1*.*3 (AV)*			
					*QSv-4B-1*.*2 (AV)*			
C7	4B-1	*D-3024409~D-1094306*	1.48	2	*QGpc-4B-1*.*4 (E2*, *AV)*	394548925~453508301	TraesCS4B01G180100~TraesCS4B01G214000	TraesCS4B01G326600LC~TraesCS4B01G376200LC
Type I					*QWa-4B-1*.*3 (AV)*			
C8	5D-1	*S-3028008~D-1401452*	7.58	2	*QBd-5D-1 (E1*, *E2*, *E3*, *AV)*	42726373~296433325	TraesCS5D01G043000~TraesCS5D01G192600	TraesCS5D01G065600LC~TraesCS5D01G263900LC
Type III					*QPv-5D-1 (E1)*			
C9	6A-2	*wPt-731556~D-3952327*	1.38	2	*QSt-6A-2*.*1 (E1*, *AV)*	50345463~50345463	-	-
Type I					*QSv-6A-2*.*1 (E1)*			
C10	6A-2	*D-1074651~D-3943958*	9.04	4	*QSt-6A-2*.*2 (E1*, *AV)*	38724043~98753555	TraesCS6A01G070900~TraesCS6A01G125200	TraesCS6A01G085000LC~TraesCS6A01G168600LC
Type I					*QSv-6A-2*.*2 (E1*, *AV)*	594529638~595926048	TraesCS6A01G367900~TraesCS6A01G370900	TraesCS6A01G566500LC~TraesCS6A01G569100LC
					*QWa-6A-2 (E2*, *E3*, *AV)*			
					*QGpc-6A-2 (E1*, *AV)*			
C11	6D-2	*D-1200944~D-3938792*	5.51	4	*QSb-6D-2 (AV)*	89723576~94664263	TraesCS6D01G125900~TraesCS6D01G129500	TraesCS6D01G153500LC~TraesCS6D01G156500LC
Type II					*QFv-6D-2 (AV)*	204504285~259293430	TraesCS6D01G181300~TraesCS6D01G188800	TraesCS6D01G156500LC~TraesCS6D01G279900LC
					*QPti-6D-2 (E3*, *AV)*			
					*QTv-6D-2 (AV)*			
C12	7A-2	*S-1211213~D-1229082*	4.02	2	*QBd-7A-2 (E2*, *E3)*	707806991~720347076	TraesCS7A01G525200~TraesCS7A01G543600	TraesCS7A01G747900LC~TraesCS7A01G774900LC
Type II					*QPv-7A-2 (E2)*			

**Cluster C2**, located on chromosome 1A between two flanking markers (*BS00089894_51~wmc312*), represents a genetic distance of 6.07 cM. This corresponds to an approximate physical interval of 16.2 Mb in a region between 497.52 and 513.75 Mb, which contains 185 HC genes and 264 LC genes based on IWGSC RefSeq v 1.0 (S5 Table, Table 5). Of the candidate genes, TraesCS1A01G317500, TraesCS1A01G466100LC, TraesCS1A01G466400LC and TraesCS1A01G466500LC, which encode the high molecular weight glutenin subunit (HMW-GS), were detected in the confidence interval of cluster C2. **Cluster C3**, located on chromosome 1B between two flanking markers (*D-996292~D-3024514*), represents a genetic distance of 6.44 cM. This corresponds to an approximately physical interval of 32.0 Mb in a region between 532.69 and 564.65 Mb, which contains 271 HC and 452 LC genes (S5 Table, Table 5). Three HMW-GS genes, including TraesCS1B01G330000, TraesCS1B01G569900LC, and TraesCS1B01G570600LC, were detected in the confidence interval of cluster C3. **Cluster C4**, located on chromosome 1D between two flanking markers (*swes1148~Ex_c68201_1182*), represents a genetic distance of 10.97 cM. This corresponds to an approximately physical interval of 28.1 Mb in a region between 407.81 and 435.94 Mb, which contains 373 HC and 444 LC genes (S5 Table, Table 5). A total of three candidate genes related to the functions of the HMW-GS were predicted on chromosome 1D, including TraesCS1D01G317300, TraesCS1D01G435600LC, and TraesCS1D01G435700LC. In the present study, the SNP markers *BS00022768_51* and *BS00082503_51*, which are tightly linked with *QSv-1A-1*, were further identified in the coding region of the TraesCS1D01G317300 and TraesCS1D01G435700LC genes, respectively. According to the BLASTN program from the National Center for Biotechnology Information nucleotide sequence database (http://blast.ncbi.nlm.nih.gov), the candidate genes TraesCS1A01G317500, TraesCS1B01G330000, and TraesCS1D01G317300 were consistent with the globulin loci reported by Gu et al. [50]; TraesCS1A01G466100LC and TraesCS1A01G466500LC were consistent with the 1Ax and 1Ay null genotpyes of the *Glu-A1* locus [50, 51], respectively; TraesCS1B01G570600LC was consistent with the y9 subunit of the *Glu-B1* locus [52]; and TraesCS1D01G435600LC and TraesCS1D01G435700LC were consistent with the x5 and y10 subunits of the *Glu-D1* locus [53, 54]. Numerous studies have identified associations between the HMW-GS and the quality of wheat [2; 16; 17; 23; 25; 26; 55; 56]. Furthermore, many previous studies have reported that QTLs for some quality traits (such as sedimentation value, development time, and stability time) were detected on the *Glu-1* loci, for example *Glu-A1* [30, 31], *Glu-B1* [17], and *Glu-D1* [12, 24, 30, 31]. In the present study, clusters C2 and C4 were determined to be colocations of stable QTLs for the SV and farinograph parameter (ST and DT) traits in more than three environments. Cluster C3 on chromosome 1B was determined to be a colocation of stable QTLs for the GPC traits under E3/AV environments. This result suggests the reliability of QTL mapping using our high-density genetic map and helps us to facilitate further fine-scale mapping and discovery of candidate genes with other QTL clusters.

In addition, we found that **clusters C5 and C6**, located on chromosome 4B, had colocalized stable QTLs for both the protein- and starch-related traits (e.g., GPC, SV, farinograph parameters and starch pasting properties) in one or more environments. For these two clusters, there was a genetic distance of 2.32/4.92 cM (for cluster C5/C6) corresponding to two approximate physical intervals of 1.7/5.0 Mb in a region between 14.65 and 16.39 Mb and 25.83 and 30.86 Mb, which contain 17/78 HC genes and 26/74 LC genes based on IWGSC RefSeq v1.0 (S5 Table, Table 5). These results may provide a molecular foundation for the annotation of QTL and QTL clusters. Moreover, we found another **cluster C10**, located on chromosome 6A between two flanking markers (*D-1074651~D-3943958*), representing a genetic distance of 9.04 cM. This corresponds to two approximate physical intervals of 60.0/1.4 Mb region between 38.72 and 98.75 Mb and 594.53 and 595.93 Mb, which contain 541/31 HC genes and 841/27 LC genes based on IWGSC RefSeq v1.0 (S5 Table, Table 5). Cluster C16 was detected based on the colocation of stable QTLs for the GPC, WA, SV, ST traits in more than two environments. However, there were no known quality genes, such as α- and β- gliadins (*Gli-A2*, *Gli-B2* and *Gli-D2*), detected based on the IWGSC_RefSeq_Annotations_v1.0 (https://urgi.versailles.inra.fr/). The α- and β- gliadins (*Gli-A2*, *Gli-B2* and *Gli-D2*) are located on the short arms of group 6 chromosomes and it has been speculated that there may be new genes for protein-related metabolism between the physical intervals of cluster C10. Therefore, these regions may facilitate further fine-scale mapping and the discovery of candidate genes. Otherwise, SNP or DArT markers closely linked to quality traits and identified by QTL mapping can be successfully converted into assays for marker-assisted breeding in the future.

## Conclusions

In this study, we used a high-density genetic map containing 10,739 loci (5,399 unique loci) of TL-RILs population to identify QTLs associated with grain protein content, sedimentation value, farinograph parameters, falling number and the performance of the starch pasting properties in wheat. A total of 106 QTLs for 13 quality traits were detected. Of these, 38 and 68 QTLs for protein- and starch-related traits were detected in three environments and the average values (AV), respectively. Twenty-six relatively high-frequency QTLs (RHF-QTLs) that were detected in more than two environments. Twelve stable QTL clusters containing at least one RHF-QTL were detected and classified into three types: type I, type II, and type III. A total of 339 markers flanked with 11 QTL clusters (all except C6), were found to be highly homologous with 282 high confidence (HC) and 57 low confidence (LC) candidate genes based on IWGSC RefSeq v1.0. Our findings might be of great usefulness for marker-assisted breeding, and could provide detailed information for the QTL fine-mapping and candidate gene discovery.

## Supporting information

S1 Fig The frequency distributions of 13 quality traits among the RILs derived from “Tainong 18 × Linmai 6” under E1, E2, E3 and AV. (DOCX)

S1 Table High density genetic map for the RILs derived from the cross “Tainong 18×Linmai 6”. (XLSX)

S2 Table The minimum LOD score for 13 quality traits detected in E1, E2, E3 and AV environments. (DOCX)

S3 Table QTLs for 13 quality traits detected in E2, E2, E3 and AV environments. (DOCX)

S4 Table Predicted candidate genes corresponding to the DArT/SNPs markers associated to the QTL clusters for quality traits based on IWGSC RefSeq v1.0. (XLSX)

S5 Table Blast results for aligning all the markers sequence from QTL cluster confidence interval with the wheat reference genome (IWGSC RefSeq v1.0). (XLSX)
