# Supplementary material for: QTL mapping for quality traits using a high-density genetic map of wheat
Source: PLoS One. 2020 Mar 24;15(3):e0230601. doi: 10.1371/journal.pone.0230601 (PMC7092975; doi:10.1371/journal.pone.0230601)
Supplement: S1 Fig — (DOCX) [file pone.0230601.s001.docx]

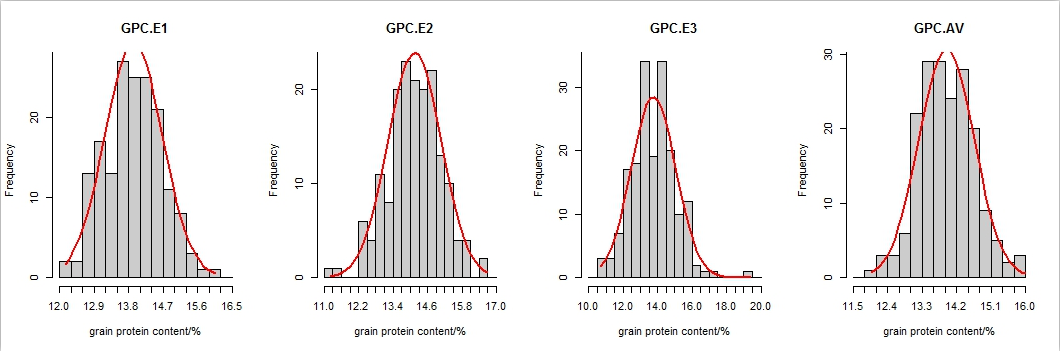


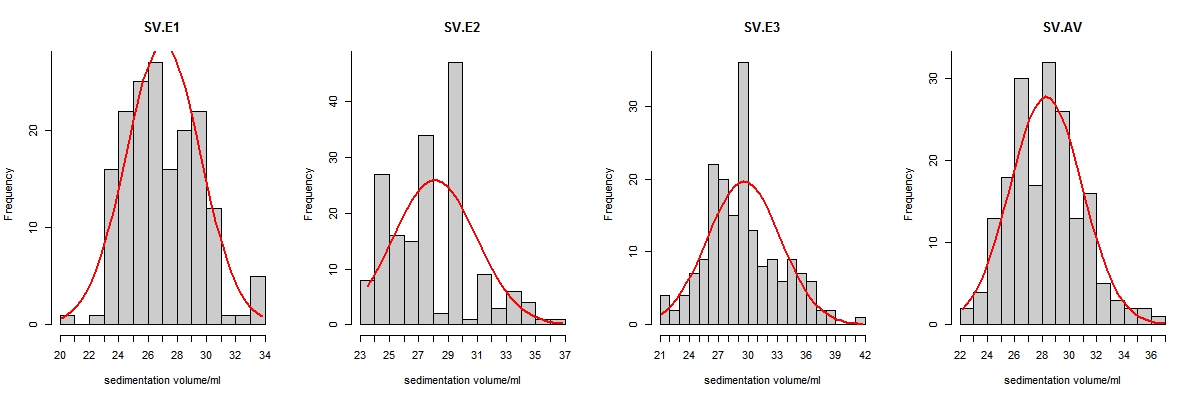


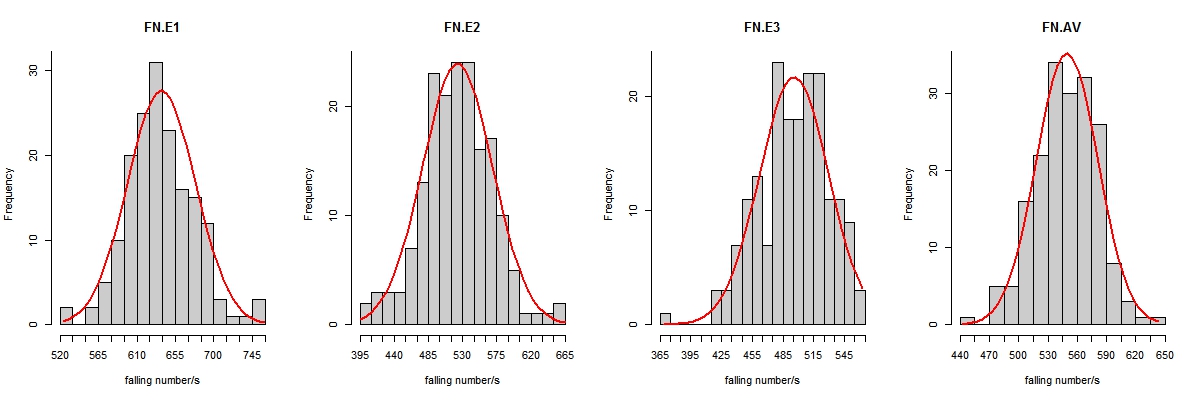


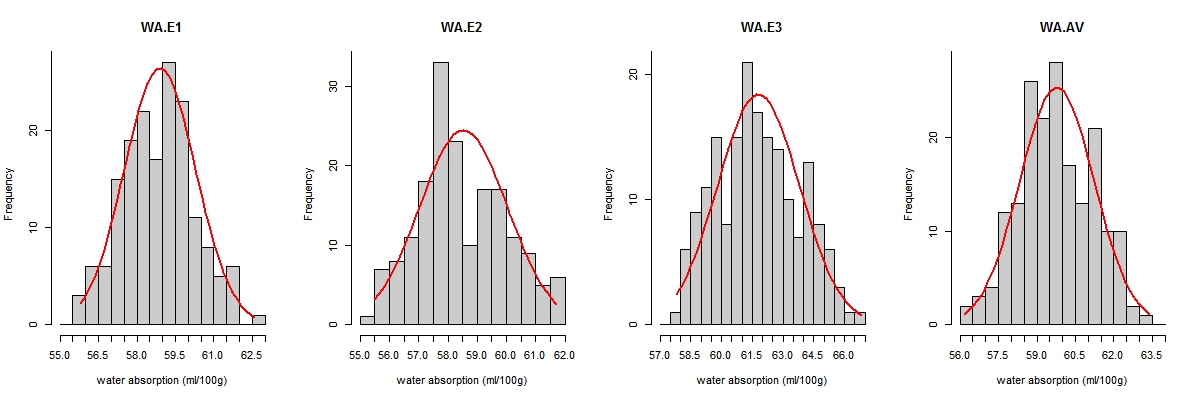


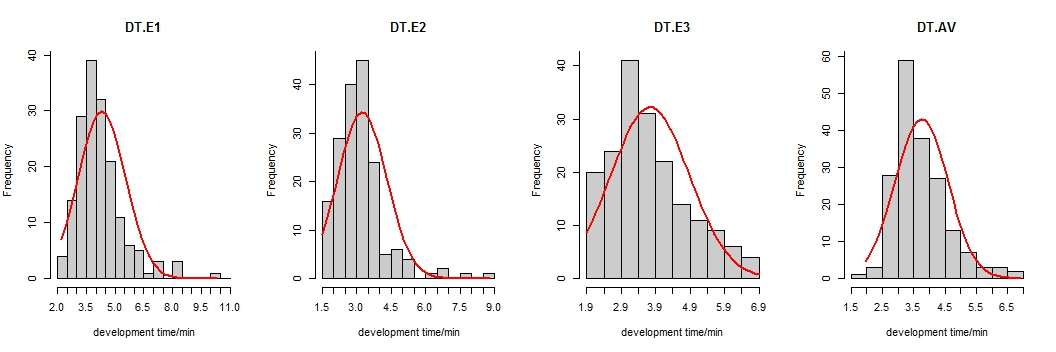


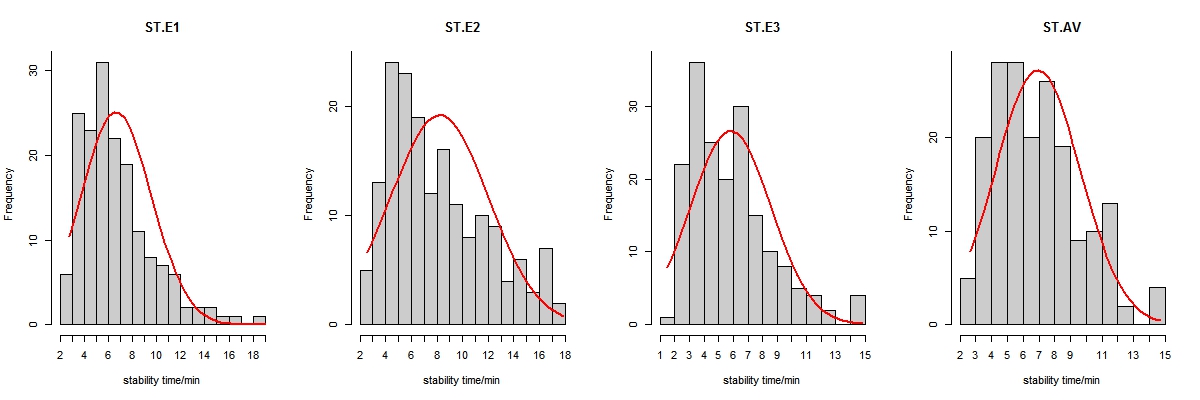


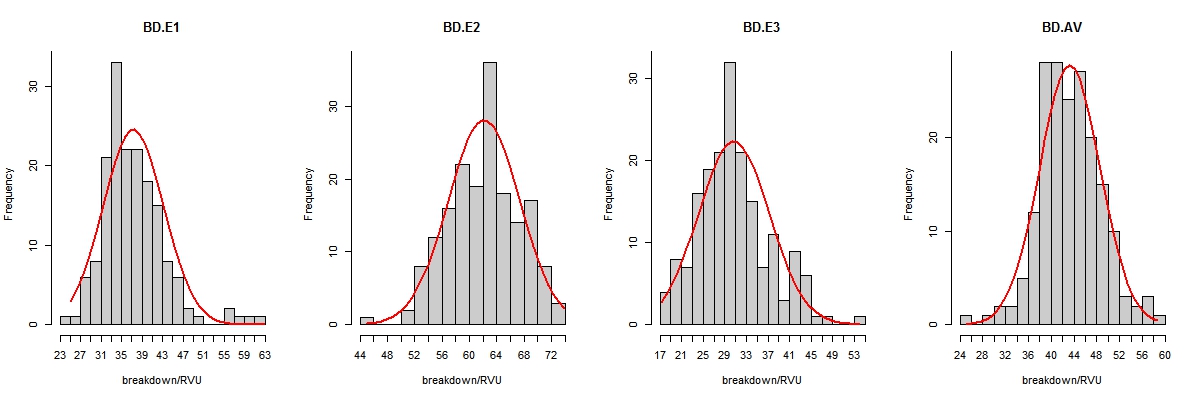


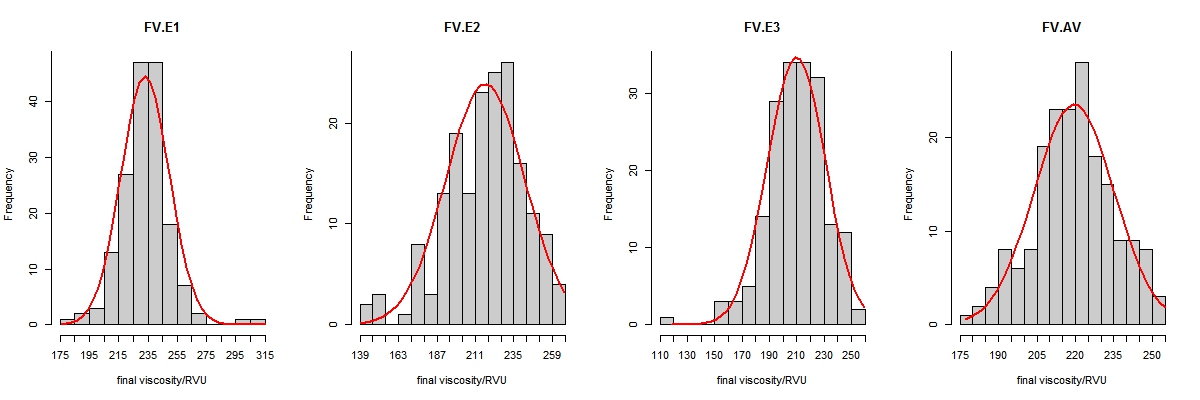


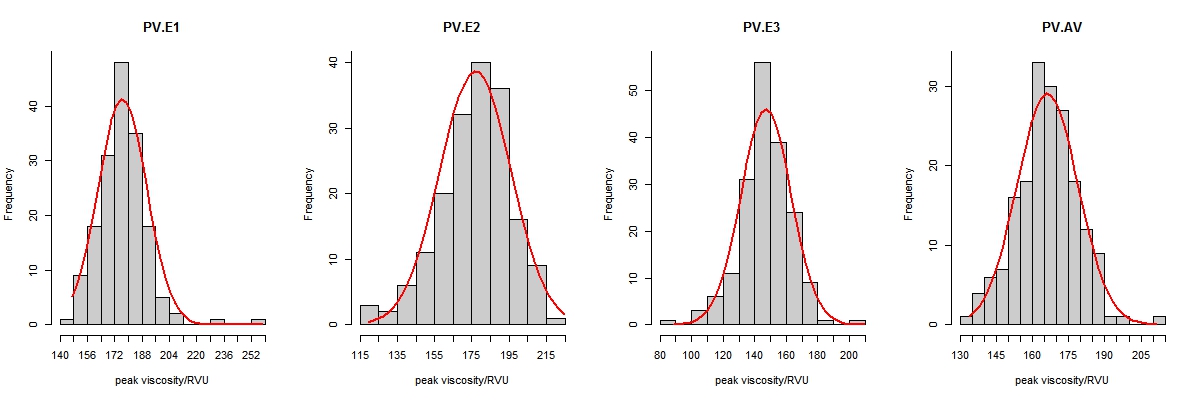


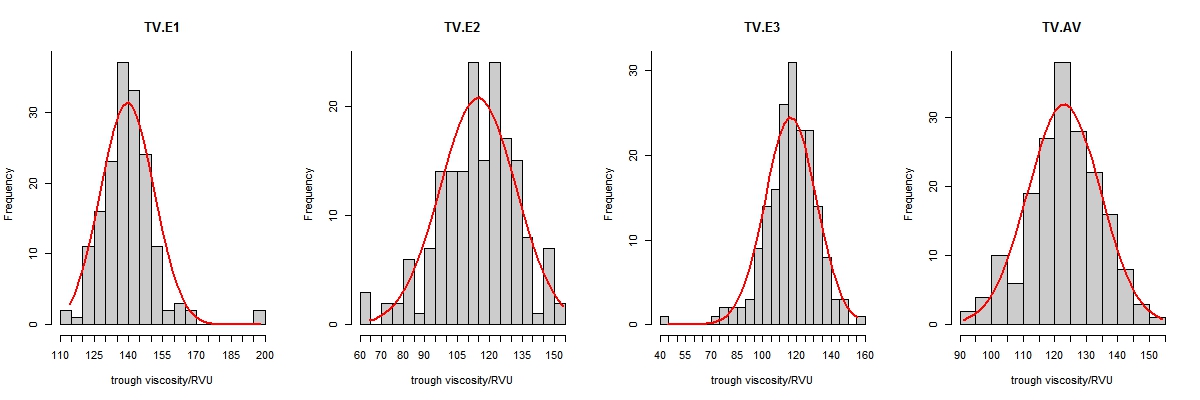


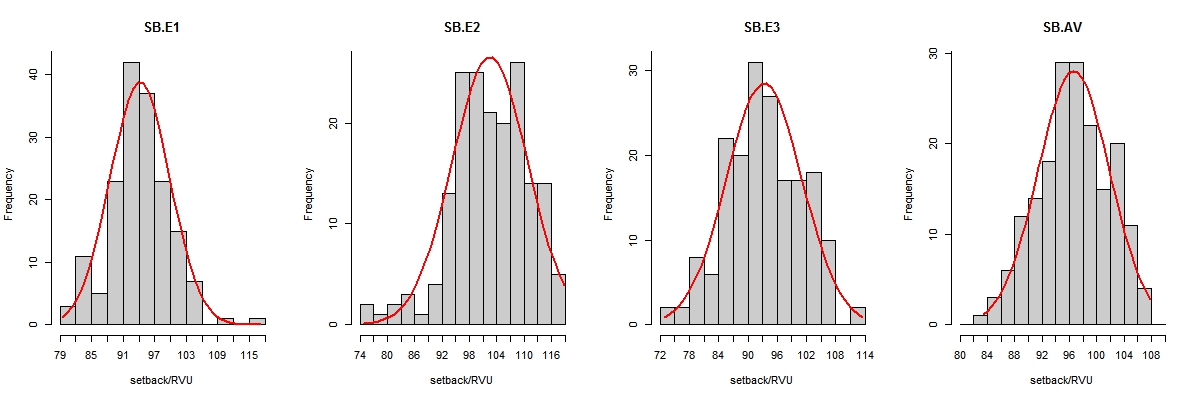

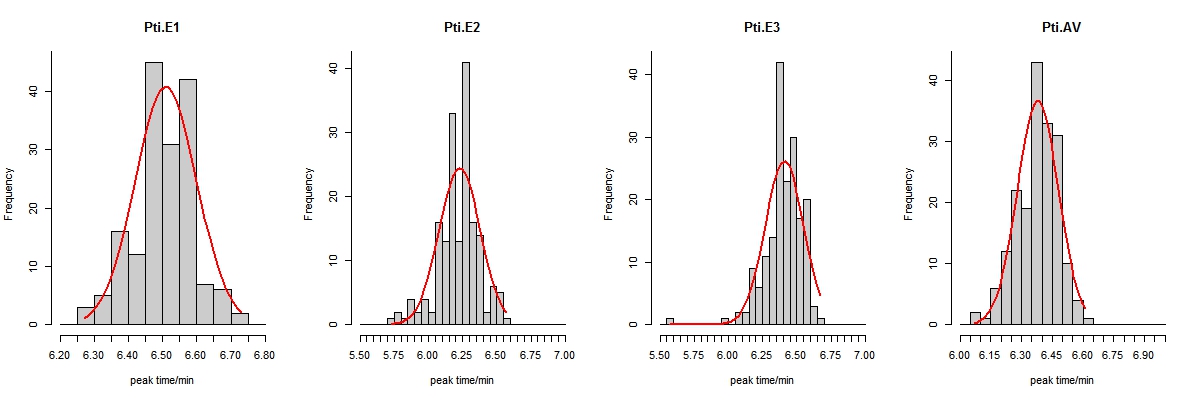

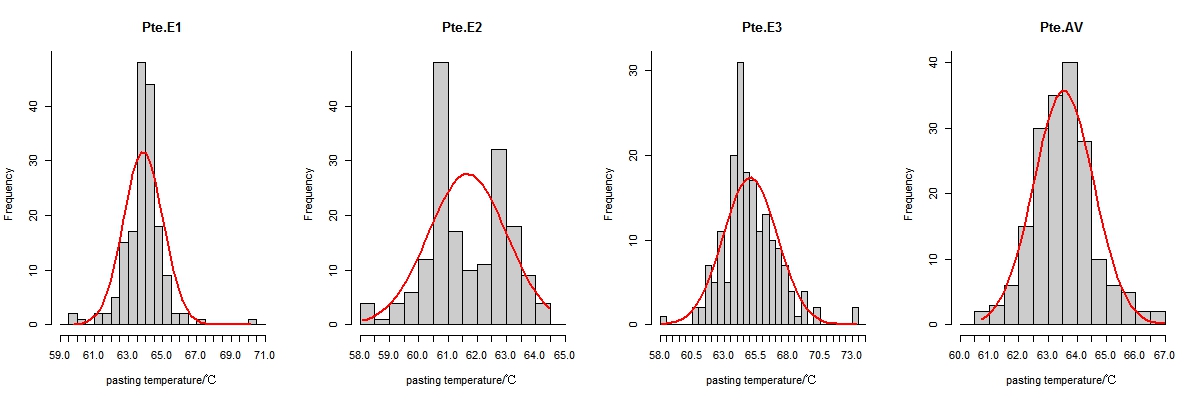


**S1 Figure. The frequency distributions of 13 quality traits among the RILs derived from “Tainong 18 × Linmai 6” under E1, E2, E3 and AV.**

(GPC, grain protein content; SV, sedimentation volume; FN, falling number; WA, water absorption; DT, development time; ST, stability time; PV, peak viscosity; TV, trough viscosity; BD, breakdown; FV, final viscosity; SB, setback; PTi, peak time; PTe, pasting temperature)
