# Supplementary material for: QTL mapping for quality traits using a high-density genetic map of wheat
Source: PLoS One. 2020 Mar 24;15(3):e0230601. doi: 10.1371/journal.pone.0230601 (PMC7092975; doi:10.1371/journal.pone.0230601)
Supplement: S2 Table — (DOCX) [file pone.0230601.s003.docx]

**S2 Table. The minimum LOD score for 13 quality traits detected in E1, E2, E3 and AV environments**

| Trait | Environment | Minimum LOD |  | Trait | Environment | Minimum LOD |
| --- | --- | --- | --- | --- | --- | --- |
| GPC | E1 | 3.9 |  | TV | E1 | 3.6 |
|  | E2 | 3.9 |  |  | E2 | 3.9 |
|  | E3 | 3.9 |  |  | E3 | 3.8 |
|  | AV | 3.9 |  |  | AV | 3.8 |
| SV | E1 | 4.0 |  | FV | E1 | 3.6 |
|  | E2 | 4.0 |  |  | E2 | 3.9 |
|  | E3 | 3.9 |  |  | E3 | 3.9 |
|  | AV | 3.9 |  |  | AV | 3.8 |
| DT | E1 | 3.6 |  | BD | E1 | 3.7 |
|  | E2 | 3.6 |  |  | E2 | 3.9 |
|  | E3 | 3.9 |  |  | E3 | 3.8 |
|  | AV | 3.9 |  |  | AV | 3.9 |
| ST | E1 | 3.9 |  | SB | E1 | 3.9 |
|  | E2 | 4.0 |  |  | E2 | 3.8 |
|  | E3 | 3.9 |  |  | E3 | 3.8 |
|  | AV | 3.9 |  |  | AV | 3.9 |
| WA | E1 | 3.9 |  | Pti | E1 | 3.9 |
|  | E2 | 4.0 |  |  | E2 | 3.8 |
|  | E3 | 3.6 |  |  | E3 | 3.8 |
|  | AV | 3.8 |  |  | AV | 3.9 |
| FN | E1 | 4.0 |  | Pte | E1 | 3.5 |
|  | E2 | 3.9 |  |  | E2 | 3.8 |
|  | E3 | 4.0 |  |  | E3 | 3.7 |
|  | AV | 4.0 |  |  | AV | 3.8 |
| PV | E1 | 3.6 |  |  |  |  |
|  | E2 | 3.8 |  |  |  |  |
|  | E3 | 3.8 |  |  |  |  |
|  | AV | 3.8 |  |  |  |  |
