# Supplementary material for: QTL mapping for quality traits using a high-density genetic map of wheat
Source: PLoS One. 2020 Mar 24;15(3):e0230601. doi: 10.1371/journal.pone.0230601 (PMC7092975; doi:10.1371/journal.pone.0230601)
Supplement: S3 Table — (DOCX) [file pone.0230601.s004.docx]

**S3 Table. QTLs for 13 quality traits detected in E1, E2, E3 and AV environments**

| **Traits ^a^** | **QTLs** | **Treatments** | **Marker interval *^b^*** | **LODs** | **Additive effects *^c^*** | ***R^2^* (%)** |
| --- | --- | --- | --- | --- | --- | --- |
| GPC | *QGpc-1B-2* | E3 | *D-1190331~S-3222160* | 7.6 | 0.5269 | 15.52 |
|  |  | AV | *D-1190331~S-3222160* | 5.5 | 0.2310 | 9.47 |
|  | *QGpc-3B-3.1* | E2 | *D-3938840~D-1261877* | 3.9 | -0.2564 | 7.08 |
|  | *QGpc-3B-3.2* | E2 | *D-1159824~D-1093997* | 4.4 | -0.2635 | 7.85 |
|  | *QGpc-4A-1* | AV | *D-1092563~IAAV3080* | 4.7 | -0.2150 | 8.69 |
|  | *QGpc-4B-1.1* | E3 | *S-3025233~D-1108965* | 4.4 | 0.3957 | 8.27 |
|  | *QGpc-4B-1.2* | E1 | *D-1113185~Ku_c63300_1309* | 5.3 | 0.2290 | 8.68 |
|  | *QGpc-4B-1.3* | AV | *D-1138250~D-3943712* | 5.2 | 0.2284 | 9.40 |
|  | *QGpc-4B-1.4* | E2 | *D-1380792~D-1094306* | 5.3 | 0.2932 | 9.82 |
|  |  | AV | *D-1380792~D-1094306* | 7.3 | 0.2756 | 14.09 |
|  | *QGpc-4D-1* | E1 | *S-1297707~Kukri_c444_833* | 4.9 | -0.2550 | 10.40 |
|  | *QGpc-6A-2* | E1 | *D-1112857~S-2362461* | 6.2 | -0.2532 | 10.66 |
|  |  | AV | *D-3948556~S-978615* | 5.7 | -0.3037 | 9.63 |
| SV | *QSv-1A-1* | E1 | *S-1089822~S-3953635* | 10.3 | 1.0387 | 16.31 |
|  |  | E2 | *D-2288948~D-1140854* | 10.3 | 1.0588 | 13.54 |
|  |  | E3 | *S-1092278~D-3022004* | 3.9 | 0.9293 | 5.71 |
|  |  | AV | *S-1089822~S-3953635* | 8.8 | 0.9003 | 10.97 |
|  | *QSv-1A-2* | E1 | *IAAV3217~swes226b* | 4.1 | -0.6855 | 6.95 |
|  | *QSv-1D-1* | E1 | *D-1234123~S-992157* | 3.9 | 0.6057 | 5.45 |
|  |  | E2 | *S-2261832~D-3384752* | 4.7 | 0.7129 | 5.88 |
|  |  | E3 | *S-992157~wsnp_Ra_c59303_60669172* | 9.5 | 1.5855 | 17.16 |
|  |  | AV | *D-1683089~S-1225816* | 10.4 | 0.9405 | 12.00 |
|  | *QSv-4B-1.1* | E3 | *S-1040960~D-1083795* | 4.6 | 0.9884 | 6.76 |
|  |  | AV | *S-1040960~D-1083795* | 5.2 | 0.6630 | 6.15 |
|  | *QSv-4B-1.2* | AV | *D-1138250~D-3943712* | 4.0 | 0.6187 | 5.32 |
|  | *QSv-5B-1* | E2 | *S-1112236~D-1229076* | 4.8 | -0.7050 | 6.06 |
|  | *QSv-5D-2* | AV | *S-985159~S-1010833* | 4.5 | -0.6315 | 5.54 |
|  | *QSv-6A-2.1* | E1 | *D-1375327~D-3952327* | 7.8 | -0.9570 | 13.34 |
|  | *QSv-6A-2.2* | E1 | *D-1112857~D-1092061* | 10.7 | -1.0493 | 16.75 |
|  |  | AV | *D-4008398~D-1092061* | 4.6 | -0.6668 | 5.40 |
|  | *QSv-6D-2.2* | E2 | *D-1107562~D-3020790* | 13.6 | -1.2772 | 18.73 |
|  | *QSv-6D-2.1* | E3 | *S-1023247~D-4329585* | 4.4 | -0.9894 | 6.65 |
|  |  | AV | *S-1023247~D-4329585* | 5.4 | -0.8762 | 9.71 |
| FN | *QFn-1D-1* | E1 | *cfd19~D-1072476* | 4.5 | -12.6569 | 9.73 |
|  |  | AV | *D-1255162~D-1073588* | 5.3 | -10.1489 | 9.55 |
|  | *QFn-2A-1* | E2 | *D-1022130~D-4004331* | 4.7 | -13.7484 | 8.43 |
|  | *QFn-5D-1* | AV | *D-3936114~S-1862723* | 4.2 | -10.2655 | 10.01 |
|  | *QFn-7D-2* | E2 | *D-1247149~D-1106330* | 5.9 | -15.4956 | 10.71 |

*^a^*, GPC, grain protein content; SV, sedimentation volume; FN, falling number; DT, development time; ST, stability time; WA, water absorption; BD, breakdown; FV, final viscosity; PV, peak viscosity; TV, trough viscosity; PTi, peak time; PTe, pasting temperature; SB, setback.

^b^ Marker interval means the interval of the LOD peak value for QTLs.

*^c^*, Positive effect, increased effect contributed by TN18; negative effect was contributed by LM6.

**S3 Table Continued-1**

| **Traits** | **QTLs** | **Treatments** | **Marker interval** | **LODs** | **Additive effects** | ***R^2^* (%)** |
| --- | --- | --- | --- | --- | --- | --- |
| ***dough rheological properties*** | | |  |  |  |  |
| DT | *QDt-1A-1* | E1 | *D-3022884~wmc312* | 8.5 | 0.4685 | 14.01 |
|  |  | E3 | *S-1000608~Kukri_c37726_285* | 5.9 | 0.3752 | 10.62 |
|  |  | AV | *S-1000608~Kukri_c37726_285* | 6.9 | 0.2948 | 11.71 |
|  | *QDt-1D-1* | E1 | *D-1170288~D-2256216* | 7.8 | 0.4493 | 12.53 |
|  |  | E3 | *D-3956372~D-2256216* | 5.1 | 0.3692 | 9.46 |
|  | *QDt-2D-2* | E1 | *D-3533182~cfd62* | 3.9 | -0.3021 | 5.89 |
|  | *QDt-6A-2* | AV | *D-1219492~D-1005732* | 4.0 | -0.2290 | 7.23 |
|  | *QDt-7D-3* | E1 | *D-1125600~D-1091022* | 4.1 | 0.3561 | 7.94 |
| ST | *QSt-1A-1* | E1 | *D-1207393~S-1000608* | 5.9 | 0.8208 | 7.55 |
|  |  | E2 | *D-1207393~S-1000608* | 12.0 | 1.5287 | 15.60 |
|  |  | E3 | *S-1092278~D-3022004* | 5.0 | 0.7335 | 6.78 |
|  |  | AV | *D-1207393~S-1000608* | 19.0 | 1.2081 | 18.84 |
|  | *QSt-1D-1* | E1 | *D-1122483~S-992228* | 16.5 | 1.5335 | 24.85 |
|  |  | E2 | *D-2309352~D-1046556* | 18.7 | 2.0563 | 26.31 |
|  |  | E3 | *D-1170288~D-2256216* | 18.2 | 1.5508 | 29.60 |
|  |  | AV | *D-1122483~S-992228* | 30.2 | 1.6344 | 35.09 |
|  | *QSt-6A-2.1* | E1 | *D-1089151~D-1076469* | 4.6 | -0.9568 | 9.21 |
|  |  | AV | *wPt-731556~D-3952327* | 10.4 | -0.8538 | 9.39 |
|  | *QSt-6A-2.2* | E1 | *D-1112857~D-1160022* | 4.9 | -0.7395 | 6.27 |
|  |  | AV | *BS00066274_51~D-1219492* | 7.1 | -0.7325 | 7.23 |
|  | *QSt-7A-1* | E1 | *wPt-3523~Kukri_c14765_1655* | 4.2 | 0.7087 | 5.65 |
| WA | *QWa-1B-3* | E1 | *D-1265604~D-3958178* | 8.6 | -0.5438 | 14.33 |
|  |  | E2 | *D-1134712~S-1027932* | 12.9 | -0.7119 | 20.37 |
|  |  | AV | *D-1265604~S-1045055* | 6.2 | -0.4630 | 7.87 |
|  | *QWa-4A-1.1* | E1 | *D-1092563~D-4329307* | 6.4 | -0.4798 | 11.82 |
|  | *QWa-4A-1.2* | E1 | *D-1159667~D-2291314* | 5.0 | -0.3841 | 7.42 |
|  | *QWa-4B-1.1* | E1 | *D-3022151~S-1040960* | 11.4 | 0.7305 | 27.02 |
|  |  | E3 | *D-3022151~S-1040960* | 10.2 | 1.0124 | 19.51 |
|  |  | AV | *D-3022151~S-1040960* | 15.7 | 0.8212 | 28.76 |
|  | *QWa-4B-1.2* | E3 | *D-1138250~D-3943712* | 5.2 | 0.8123 | 9.21 |
|  |  | AV | *D-1138250~D-3943712* | 10.4 | 0.7523 | 18.13 |
|  | *QWa-4B-1.3* | AV | *D-1380792~D-1094306* | 5.1 | 0.4353 | 6.79 |
|  | *QWa-6A-2* | E2 | *D-1026376~S-2362461* | 5.4 | -0.4153 | 7.47 |
|  |  | E3 | *D-1083065~D-3384829* | 5.4 | -0.6631 | 8.31 |
|  |  | AV | *D-1095649~Excalibur_rep_c69981_75* | 6.7 | -0.4917 | 10.34 |

**S3 Table Continued-2**

| **Traits** | **QTLs** | **Treatments** | **Marker interval** | **LODs** | **Additive effects** | ***R^2^* (%)** |
| --- | --- | --- | --- | --- | --- | --- |
| ***starch pasting properties*** | | |  |  |  |  |
| BD | *QBd-1B-2* | AV | *D-996292~D-3384667* | 5.4 | -1.6589 | 9.51 |
|  | *QBd-1D-1* | E2 | *S-1094719~wmc429* | 4.1 | 1.9843 | 14.05 |
|  | *QBd-5A-1* | E2 | *S-3022014~D-3937104* | 4.5 | 1.5498 | 8.60 |
|  | *QBd-5D-1* | E1 | *S-982589~S-1052739* | 6.4 | 2.1168 | 11.81 |
|  |  | E2 | *D-3948993~D-1055236* | 4.6 | 1.5379 | 8.20 |
|  |  | E3 | *S-1862723~S-1081384* | 9.0 | 2.6700 | 15.96 |
|  |  | AV | *D-1687765~D-3957770* | 7.9 | 1.9207 | 12.80 |
|  | *QBd-6B* | E1 | *D-3020692~S-1054930* | 5.6 | -1.9717 | 10.64 |
|  |  | AV | *D-1100407~S-1031850* | 6.3 | -1.7621 | 10.57 |
|  | *QBd-7A-2* | E2 | *D-3575352~D-1127555* | 4.7 | -1.5677 | 8.34 |
|  |  | E3 | *S-987549~D-1107370* | 6.9 | -4.0067 | 13.48 |
|  | *QBd-7D-3* | E3 | *S-1199619~S-1206653* | 4.1 | 3.0819 | 7.27 |
|  | *QBd-7D-3* | E1 | *wPt-732048~D-1072335* | 5.1 | -1.8725 | 9.33 |
|  |  | AV | *wPt-732048~D-1072335* | 6.2 | -1.6852 | 9.67 |
| FV | *QFv-1A-1* | E2 | *D-3385037~wPt-732946* | 6.9 | 8.5222 | 11.46 |
|  |  | AV | *D-1071305~S-3030753* | 3.8 | 4.0388 | 6.31 |
|  | *QFv-2B-1* | AV | *S-1045872~S-1060973* | 6.8 | 6.6600 | 11.93 |
|  | *QFv-2B-2* | E2 | *D-1162944~D-4329591* | 4.2 | 6.4850 | 6.75 |
|  | *QFv-4B-1.1* | E1 | *D-1003776~D-2322374* | 4.5 | 5.6297 | 8.90 |
|  | *QFv-4B-1.2* | E1 | *D-3939025~D-983005* | 6.5 | -6.9636 | 13.27 |
|  | *QFv-4B-1* | AV | *D-4008856~D-1138250* | 4.0 | -4.1980 | 6.59 |
|  | *QFv-6D-2* | AV | *D-2265140~D-3938792* | 5.8 | -5.0501 | 9.59 |
|  | *QFv-7D-2.1* | E2 | *D-1309290~D-1106330* | 6.3 | -8.0998 | 10.29 |
|  | *QFv-7D-2.2* | E2 | *D-2255723~BS00049220_51* | 4.8 | -8.0078 | 10.32 |
| Pte | *QPte-1A-1* | E3 | *BS00105601_51~D-3942600* | 4.3 | 0.6911 | 9.07 |
|  | *QPte-1B-2* | E2 | *D-1139613~D-1145391* | 4.5 | -0.4121 | 9.21 |
|  | *QPte-1D-1* | E3 | *D-1170288~D-2256216* | 4.0 | -0.6253 | 7.88 |
|  | *QPte-3B-3* | E2 | *D-1150164~S-987924* | 4.1 | 0.4025 | 8.53 |
|  | *QPte-5B-1* | AV | *D-3943900~D-1151694* | 4.4 | -0.3222 | 8.59 |
|  | *QPte-7D-2* | E2 | *D-2252091~D-2369933* | 4.8 | -0.4463 | 10.28 |
| Pti | *QPti-1A-1.1* | E2 | *wPt-734027~D-1101586* | 5.0 | 0.0448 | 8.17 |
|  | *QPti-1A-1.2* | E2 | *D-1283455~RFL_Contig1118_65* | 4.6 | 0.0548 | 10.59 |
|  |  | AV | *D-1283455~RFL_Contig1118_65* | 5.5 | 0.0323 | 9.84 |
|  | *QPti-1A-1* | E2 | *S-1126925~wsnp_CAP12_c2438_1180601* | 5.1 | 0.0456 | 8.54 |
|  | *QPti-1B-2* | E1 | *D-1092345~D-2280958* | 4.0 | 0.0254 | 8.10 |
|  | *QPti-2B-1* | AV | *S-1045872~S-1060973* | 10.6 | 0.0548 | 17.90 |
|  | *QPti-2B-2* | E2 | *D-3020753~D-3950649* | 4.8 | 0.0437 | 8.15 |
|  | *QPti-6B* | E1 | *D-1082983~D-1127421* | 4.0 | 0.0245 | 7.64 |
|  | *QPti-6D-2* | E3 | *D-1129747~D-2265140* | 5.4 | -0.0471 | 11.07 |
|  |  | AV | *D-2265140~D-3938792* | 4.1 | -0.0261 | 6.30 |
|  | *QPti-7D-2* | E2 | *D-1247149~D-1106330* | 7.1 | -0.0526 | 12.22 |

**S3 Table Continued-3**

| **Traits** | **QTLs** | **Treatments** | **Marker interval** | **LODs** | **Additive effects** | ***R^2^* (%)** |
| --- | --- | --- | --- | --- | --- | --- |
| ***starch pasting properties*** | | |  |  |  |  |
| PV | *QPv-2A-2* | E2 | *D-1864258~D-3960089* | 4.9 | 5.6752 | 8.36 |
|  | *QPv-4B-1.1* | AV | *D-1117674~D-3953244* | 4.4 | -3.7038 | 8.15 |
|  | *QPv-4B-1.2* | E3 | *D-4008856~D-1138250* | 3.9 | -4.5129 | 7.47 |
|  | *QPv-5D-1* | E1 | *S-1052739~D-3950436* | 5.5 | 4.8506 | 11.26 |
|  | *QPv-7A-1* | E1 | *D-1108743~S-1042718* | 4.2 | -4.2616 | 8.70 |
|  | *QPv-7A-2* | E2 | *S-1211213~D-1249601* | 3.8 | -4.9955 | 6.54 |
|  | *QPv-7D-2.1* | E2 | *D-3946989~D-1106330* | 6.9 | -6.9286 | 12.39 |
|  | *QPv-7D-2.2* | E2 | *D-2255723~BS00049220_51* | 4.5 | -6.2930 | 10.58 |
| SB | *QSb-1A-1* | E2 | *D-3385037~wsnp_CAP12_c2438_1180601* | 5.1 | 2.4484 | 8.50 |
|  | *QSb-1B-2* | AV | *D-3533876~D-1112014* | 5.1 | -1.5397 | 8.16 |
|  | *QSb-2A-1* | E2 | *D-4004331~D-1281697* | 5.0 | -2.4598 | 8.48 |
|  | *QSb-2D-1* | AV | *IAAV6612~wsnp_Ex_c58019_59494143* | 6.3 | 1.7370 | 10.39 |
|  | *QSb-2D-2.1* | E2 | *D-1205723~D-3956230* | 5.8 | -2.7117 | 9.70 |
|  | *QSb-2D-2.2* | E2 | *D-1220601~D-1114318* | 4.1 | -2.4346 | 7.57 |
|  | *QSb-3A-1* | E1 | *BS00094366_51~wsnp_Ex_c15674_24005648* | 4.4 | 1.8447 | 10.27 |
|  | *QSb-3B-2* | E3 | *D-3027061~D-4329600* | 4.8 | -2.5055 | 9.73 |
|  | *QSb-3B-3* | E1 | *S-2258550~D-1081439* | 6.2 | -2.0085 | 12.30 |
|  | *QSb-4B-1.1* | AV | *D-3022151~S-1040960* | 5.5 | -1.6554 | 9.44 |
|  | *QSb-4B-1.2* | AV | *D-4008856~D-1138250* | 4.9 | -1.5719 | 8.37 |
|  | *QSb-6A-2.1* | AV | *D-3960900~S-1116215* | 4.2 | -1.4418 | 6.87 |
|  | *QSb-6A-2.2* | AV | *S-4329742~D-990466* | 5.5 | -1.6134 | 8.91 |
|  | *QSb-6D-2* | AV | *D-1200944~D-3938792* | 4.3 | -1.4994 | 7.32 |
|  | *QSb-7D-2.1* | E2 | *D-2249237~S-1093023* | 6.0 | -2.7581 | 10.74 |
|  | *QSb-7D-2.2* | E2 | *D-2252091~D-2369933* | 9.0 | -3.4594 | 15.88 |
| TV | *QTv-1A-1* | E2 | *wPt-732946~D-1071305* | 6.7 | 6.2003 | 12.01 |
|  | *QTv-1D-2* | E2 | *D-2247438~D-1062648* | 4.1 | -4.8101 | 7.06 |
|  | *QTv-2B-1* | AV | *S-1045872~S-1060973* | 7.9 | 5.3393 | 14.28 |
|  | *QTv-4B-1.1* | E1 | *D-1003776~D-1114147* | 5.1 | 4.2878 | 10.49 |
|  | *QTv-4B-1.2* | E1 | *D-983005~D-1111564* | 6.8 | -5.0687 | 14.47 |
|  | *QTv-6D-2* | AV | *D-1129747~D-2265140* | 7.0 | -4.1471 | 12.25 |
|  | *QTv-7D-2.1* | E2 | *D-1309290~D-1106330* | 6.5 | -6.0060 | 11.31 |
|  | *QTv-7D-2.2* | E2 | *D-2255723~BS00049220_51* | 4.2 | -5.7031 | 10.10 |
